# Molecular epidemiology of *Paracoccidiodes* spp. recovered from patients with paracoccidioidomycosis in a teaching hospital from Minas Gerais State of Brazil

**DOI:** 10.1371/journal.pntd.0009956

**Published:** 2021-11-29

**Authors:** Juliana Andrade-Silva, Leonardo Euripedes Andrade-Silva, Hugo Costa Paes, Lucas Alves, Adair Rosa, Bernardo Guerra Tenório, Marcelo Simão Ferreira, Maria Sueli Soares Felipe, Marcus de Melo Teixeira, Mario León Silva-Vergara

**Affiliations:** 1 Infectious Diseases Unit, Internal Medicine Department, Triangulo Mineiro Federal University, Uberaba, Brazil; 2 Faculty of Medicina, University of Brasília, Brasília, Brazil; 3 Infectious diseases Unit, Internal Medicine Department Federal University of Uberlândia, Uberlândia, Brazil; 4 Graduate Program in Genomic Sciences, Catholic University of Brasília, Brasília, Brazil; Universidad de Antioquia, COLOMBIA

## Abstract

**Introduction:**

Paracoccidioidomycosis (PCM) is caused by several species of the *Paracoccidioides* genus which can be differentiated by interspecific genetic variations, morphology and geographic distribution. Intraspecific variability correlation with clinical and epidemiological aspects of these species still remains unclear. This study aimed to sequence the loci GP43, exon 2 and ARF of 23 clinical isolates of *Paracoccidioides* spp. from patients in the Southeast Region of Brazil.

**Methodology and main findings:**

GenBank was used to compare the present (23) with previous described sequences (151) that included ARF and GP43. It was identified a high polymorphism rate among the 23 isolates in comparison to the other 151. Among the isolates, 22 (95.66%) were S1/*P*. *brasiliensis* and 1 (4.34%) was identified as PS2/*P*. *americana*. A total of 45 haplotypes were found as follows: 19 from S1/*P*. *brasiliensis* (13 from the present study), 15 from *P*. *lutzii*, 6 from PS2/*P*. *americana* (1 from the present study), 3 from PS3/*P*. *restrepiensis* and 2 from PS4/*P*. *venezuelensis*. Moreover, exclusive haplotypes according to clinical origin and geographical area were found. S1/*P*. *brasiliensis* (HD = 0.655 and K = 4.613) and *P*. *lutzii* (HD = 0.649 and K = 2.906) presented the highest rate of polymorphism among all species, from which 12 isolates of the present study were clustered within S1b/*P*. *brasiliensis*. The GP43 *locus* showed a higher variability and was found to be the main reason for the species differentiation.

**Conclusions:**

The results herein decribed show a high intraspecific genetic variability among S1/*P*. *brasiliensis* isolates and confirm the predominance of this species in the Southeast region of Brazil. The finding of exclusive haplotypes according to clinical origin and geographical area would suggest correlation between the molecular profile with the clinical form and geographic origin of patients with PCM.

## Author summary

Paracoccidioidomycosis (PCM) is one of the most important systemic mycosis of Latin America. This disease can be caused by *Paracoccidioides lutzii* and four different phylogenetic species: S1/*Paracoccidioides brasiliensis sensu stricto* that harboring S1a and S1b, PS2/*Paracoccidioides americana*, PS3/*Paracoccidioides restrepiensis* and PS4/*Paracoccidioides venezuelensis*. Some of these species show differences in their main geographic region of predominance such as PS2/*P*. *americana* that can be found in Venezuela and Southern Brazil; PS3/*P*. *restrepiensis* and PS4/*P*. *venezuelensis* which are distributed in Colombia and Venezuela. However, and due to their wide geographical distribution, the species S1/*P*. *brasiliensis* and *P*. *lutzii* overlapping ecological niches and can be found in different regions of Brazil and other Latin American countries. Regarding eco-epidemiological aspects, the habitat is believed to be the soil due to the predominance of the disease among rural workers who become infected by inhaling infectious propagules during their farm activities. According to other authors, these species could have relation with the different PCM clinical presentation. This study aimed to describe the molecular epidemiology associated with clinical and epidemiological data of *Paracoccidiodes* spp. in the Minas Gerais State, located in the Southeast region, Brazil. Among the 23 isolates herein evaluated, 22 were S1/*P*. *brasiliensis* and 1 was identified as PS2/*P*. *americana*. A total of 45 haplotypes were found when these isolates were compared with other 151 deposited in the Genbank. The preliminar finding of exclusive haplotypes according to clinical origin and geographical area would suggest correlation between the molecular profile with the clinical form and geographic origin of patients with PCM. The GP43 *locus* showed a higher variability and was found to be the main promotor of species differentiation. The results herein described pointed out a high intraspecific genetic variability among S1/*P*. *brasiliensis* isolates and confirm the predominance of this species in the Southeast region of Brazil.

## Introduction

Paracoccidioidomycosis (PCM) is caused by a thermodimorphic fungi from the *Paracoccidioides* genus and it is considered one of the most prevalent endemic-systemic mycoses in Latin America [1,2]. Nearby, 80% of all the PCM cases from Latin America are diagnosed in Brazil where it represents the 8^th^ cause of death among other chronic infectious diseases. PCM fill all criteria to be considered as a neglected nosological entity [3,4].

Classically, PCM presents two different clinical forms: acute/subacute which is commonly described in children and young adults who present severe systemic and progressive symptoms related to mononuclear phagocytic system and the skin; and the chronic ones which represents 80–90% of all cases and occurs mainly in male adults who present pulmonary and mucosal commitment [5,6]. Since 1989 when it was described the first case of PCM associated to HIV infection, over 200 patients with this coinfection have been reported. These patiens exhibited a faster development of a more aggressive clinical display, acute and chronic symptoms overlapping, and frequent systemic dissemination. Several experts have suggested a third clinical form associated with immunodeficiency, therefore named “mixed” PCM [7–9].

PCM taxonomy is constantly evolving as new technologies and approaches are introduced. As of now, *P*. *brasiliensis* complex is composed by at least 5 genetically isolated groups: S1/*P*. *brasiliensis sensu stricto* with strong population structure in Brazil and harboring S1a and S1b, two distinct populations that are found in the Midwest, Southeast and South regions of Brazil [10,11]; PS2/*P*. *americana* can be found in Venezuela and Southeastern Brazil; PS3/*P*. *restrepiensis* and PS4/*P*. *venezuelensis* overlap their distribution over Colombia and Venezuela [12–14]; and last but not least *P*. *lutzii* that comprises a single species found in Equador and Central Western/Amazonian regions of Brazil [12,13,15,16]. Divergency times among species pairs range from 0,03 to 33 million years and may be explained by geographical overlapping [10]. In addition, Brazil and Venezuela might harbor more than one species of *Paracoccidioides* which opens the possibility for gene exchange between those species [17] and therefore the emergence of new admixed species as recently described [18].

Molecular characterization of clinical isolates of *Paracoccidioides* spp. allows a better understanding of correlations involving species/genotype, geographical distribution, clinical phenotype, host preference, reinfection frequency, pathogen evolution and therapeutic response [19,20].

The present study aimed to characterize, using phylogenomic and population genetics tools, the cryptic species of *Paracoccidioides* clinical isolates recovered from patients with PCM diagnosed and treated at the teaching hospital from Universidade Federal do Triângulo Mineiro, Minas Gerais State of Brazil. This region is considered an PCM endemic zone with reports of both *P*. *brasiliensis* and *P*. *lutzii* complexes.

## Methods

### Ethics statement

All samples used in this study were retrieved from the culture collection of the Mycology Laboratory of the Triângulo Mineiro Federal University. All data were deidentified. Institutional human research ethics approval for the study was obtained from the Research Ethics Board of the Triângulo Mineiro Federal University (protocol CIBIO/UFTM 50, 18/06/2015). The need for consent was waived by the Ethics Board.

### Clinico-epidemiological data collection

The University Hospital of the Federal University of Triangulo Mineiro serves population of 27 municipalities that make up the macro Southern Triangulo Mineiro region and has an estimated coverage of one million inhabitants, which corresponds to about 11% of the total population of the State of Minas Gerais (Brazilian Institute of Geography and Statistics, 2019) [21]. Retrospectively, the medical records of patients with a diagnosis of PCM confirmed by culture and direct examination and admitted at the Infectious and Parasitic Diseases ward from 2008 to 2019 were reviewed. The most relevant demographic, epidemiological, clinical and outcome data in the context of PCM were obtained.

### Fungal isolates

Twenty three clinical isolates identified by conventional mycological methods were included in this study [22]. Isolates were obtained from the following clinical sources: 01 from cerebrospinal fluid (CSF), 4 from lymph nodes, 3 from bronchoalveolar lavage (BAL), 2 from skin fragment, and 2 from peripheral blood, 2 from lung fragment and 9 from oral lesion (S1 Table). Isolates were maintained in Fava-Netto agar tubes incubated at 37° C for yeast growth and cultivated every 30 to 60 days [23].

### DNA extraction and loci selection

Genomic DNA was extracted from yeast cells using phenol-chloroform-isoamyl alcohol method as described previously [24]. DNA quantification and integrity were measured by photometry in NanoDrop Lite, Thermo Scientific [25]. The loci ARF and GP43 were chosen for identification of *Paracoccidioides* spp. isolates since they have a most complete databank. The present 23 isolates’ genome were added to the previous 151 sequences in GenBank (https://blast.ncbi.nlm.nih.gov/Blast.cgi) (S1 Table).

### PCR of the *Paracoccidioides* spp. isolates

DNA amplifications were performed by Polymerase Chain Reaction (PCR) of coding genes for the ADP Partial Ribosylation Factor (ARF) and the 43kDa glycoprotein (gp43 –*exon* 2) using Platinum Taq DNA polymerase 2X PCR Master Mix. The mixture contained 5 μL of 10X reaction buffer solution, 1 μL of forward primers (ARF-F 5’CATGGTTGGCCTCGATGCTGCC3’, gp43-E2F 5’CCAGGAGGCGTGCAGGTGTCCC3’) and reverse (ARF-R 5’GAGCCTCGACGACACGGTCACGATC3’, gp43-E2F 5’CCAGGAGGCGTGCAGGTGTCCC3’) and reverse (ARF-R 5’GAGCCTCGACGACACGGTCACGATC3’, gp43-E2F GCCCCCTCCGTCTTCCATGTCC3’) (10 pM) previously described, 5 μL of deoxynucleoside triphosphate solution (0.2 mM), 2 μL of magnesium chloride solution (2 mM), 0.5 μL of Taq DNA polymerase (2.5 U), 100 ng of Fungal genomic DNA and ultrapure water in a final reaction volume of 50 μL. Times and temperatures conditions for cycling were adapted according to previous authors [13,26–28].

### Haplotype analysis

Genetic polymorphism analysis was performed by concatenated sequences of both ARF and GP43 loci. PCR products were purified using PCR purification KIT (250)– 28106 (QIAGEN) and submitted to Sanger sequencing. Resulting sequences were edited using the Chromas-pro v. 1.7.6 software available at http://technelysium.com.au/ChromasPro.html. In addition, only sequences with a *Phred* quality score > 20 were included to limit the possibility of incorrect nucleotide bases incorporation to 1 in 100 (99% accuracy). Consensus sequences were obtained from forward and reverse readings using Chromas-pro 1.7.6. [29]. The allele types (AT) and haplotypes (H) were identified using MLSTest 1.0 software [30].

### Phylogenetic analysis

The phylogenetic analysis was performed in MEGA 7.0 [29,31]. Consensus sequences of the isolates and those obtained from GenBank were aligned using the Clustal W2 algorithm available at https://www.ebi.ac.uk/Tools/msa/clustalw2/ [32]. The allelic sequences for each isolate were concatenated, and the evolutionary relationships, with 1000 bootstrap replicates, were inferred by construction of an unrooted maximum likelihood (ML) phylogenetic tree. In addition, the data set was subjected to neighbor joining (NJ), maximum parsimony (MP), and the unweighted pair group method with arithmetic mean (UPGMA) analysis [29]. The especies of *Paracoccidioides* were confirmed according to phylogenetic clustering with the reference type strains of each especie by construction of an unrooted maximum likelihood (ML) phylogenetic tree.

### Nucleotide diversity

DNASP 5.10 [33] was used to calculate the extent of DNA polymorphism, including the number of polymorphic sites (S), nucleotide diversity (p), number of haplotypes (h), haplotype diversity (Hd), and average number of nucleotide diferences (k). The neutrality test Tajima’s D, Fu & Li’s D*, Fu & Li’s F*, and Fu’s Fs were also calculated. Negative or positive results of these tests provide evidence of purifying or balancing selection, respectively. The Watterson estimator (theta) method was used to determine the degree of recombination within the population using DNASP 5.10. The presence of recombination was also checked by measuring the phylogenetic compatibilities of nearby polymorphic sites along single and concatenated sequences in SPLITSTREE v. 4.13.1 (https://mybiosoftware.com/splitstree-compute-phylogenetic-networks.html) [34]. This analysis was performed by applying the uncorrected (observed, ‘*P*’) distances in characters transformation using the neighbor-net algorithm [34]. The pairwise homoplasy index (PHI) was used to assess statistical significance for recombination.

### Statistical analyses

Statistical analyses were performed using DNAsp 5.10 [33] MS Excel (Microsoft Corporation) and SplitsTree v. 4.13.1 [34]. *P*-values less than 5% (*P <* 0.05) were considered statistically significant.

## Results

### Clinical and epidemiological results

Of the 23 patients with PCM evaluated, 18 (78.3%) were men, with a mean age of 37.4 years. The chronic form was characterized in 10 (43.47%) of the cases, the acute/subacute form in eight (34.83%) and the mixed form in five (21.74%) cases (Table 1). The diagnosis of PCM was confirmed in 23/23 (100%) of the cases by culture and additionally in 11/23 (47.83%) by histopathology, and in 12/23 (52.1%) by KOH direct examination. Patients with the acute and mixed forms were treated with amphotericin B followed by itraconazole, whereas patients with the chronic form received itraconazole. Of the 23 patients, 8 (34.78%) were HIV infected. Among patients co-infected with HIV, most were male 6/8 (75%), the average age was 32.9 years, five (62.5%) presented the mixed clinical form, five (62.5%) were originated from the Minas Gerais State (MG), seven (87.5%) presented S1/*P*. *brasiliensis* infection and the most common outcome was cure (75%).

**Table 1:** Main clinical and epidemiological data of 23 patients with PCM of whom isolates of *Paracoccidiodes* spp. were obtained.

Assessed Data	N° of isolates (%)
**Gender**	
Male	18 (78.3)
Female	5 (22.7)
**Age group**	
00–13	1 (4.3)
14–30	6 (26.1)
31–40	8 (34.8)
41–50	6 (26.1)
˃50	2 (8.6)
**Geographic region**	
Minas Gerais (Southeast)	17 (73.9)
Goiás (Midwest)	1 (4.3)
São Paulo (Southeast)	5 (21.7)
**Clinical Form**	
Acute/Subacute	8 (34.8)
Chronic	10 (43.5)
Mixed (HIV associated)	5 (21.7)
**Recurrence of PCM**	7 (30.4)
Male	4 (57.4)
Female	3 (42.6)
**Death**	3 (13.0)
Acute/Subacute	1 (33.3)
Mixed	2 (66.7)
**HIV (Co-infection)**	8 (34.8)
Male	6 (75)
Female	2 (25)

PCM—paracoccidioidomycosis

### Haplotype diversity (GP43+ARF)

**Fig 1:**
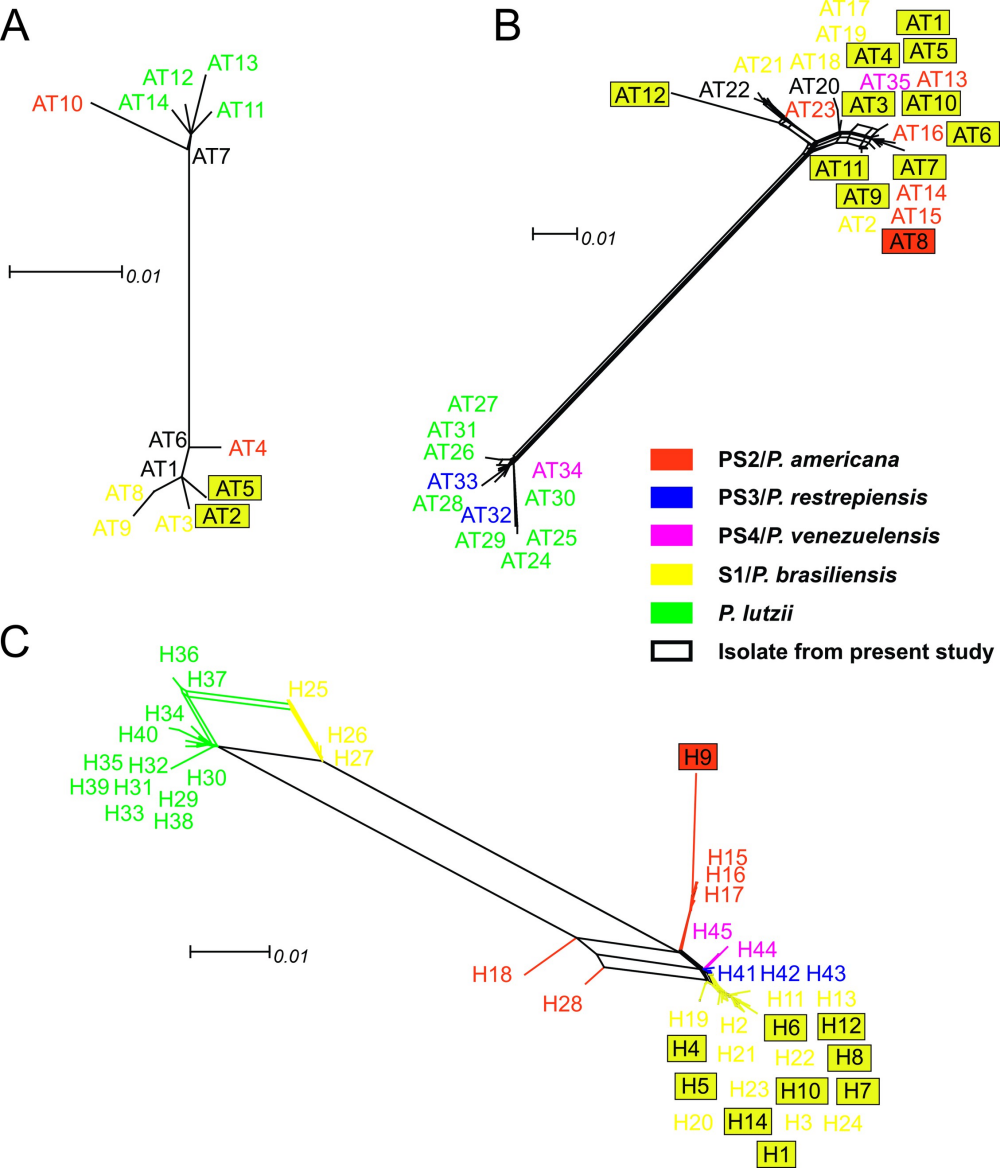
**Split decomposition analysis of the locus ARF (A), locus GP43 (B) and concatenated loci ARF + GP43 (C) of the 174 *Paracoccidioides* spp. isolates applying the neighbournet algorithm by means of the uncorrect-P parameter model to evidence the diversity and branching ambiguities attributable to recombination events.** The observation that isolates are linked to each other by multiple pathways and are forming an interconnected network rather than a single bifurcating tree is suggestive of recombination. The phi test for recombination implemented in the software SplitsTree showed significant evidence (p*<*0.0001) for recombination in the ARF+GP43. In the single locus evaluated are demonstred the allele types (ATs). In the concatenated sequences are demonstred the haplotipes (H) found. The especies are diferenciated by colors as follow: red PS2/*P*. *americana*, blue PS3/*P*. *restrepiensis*, pink PS4/*P*. *venenzuelensis*, yellow S1/*P*. *brasiliensis* and green *P*. *lutzii*. The exclusives ATs and H of a specific species are shown by the color indicative of the species. The exclusives ATs and H of isolates from present study are marqued by a frame.

**Fig 2:**
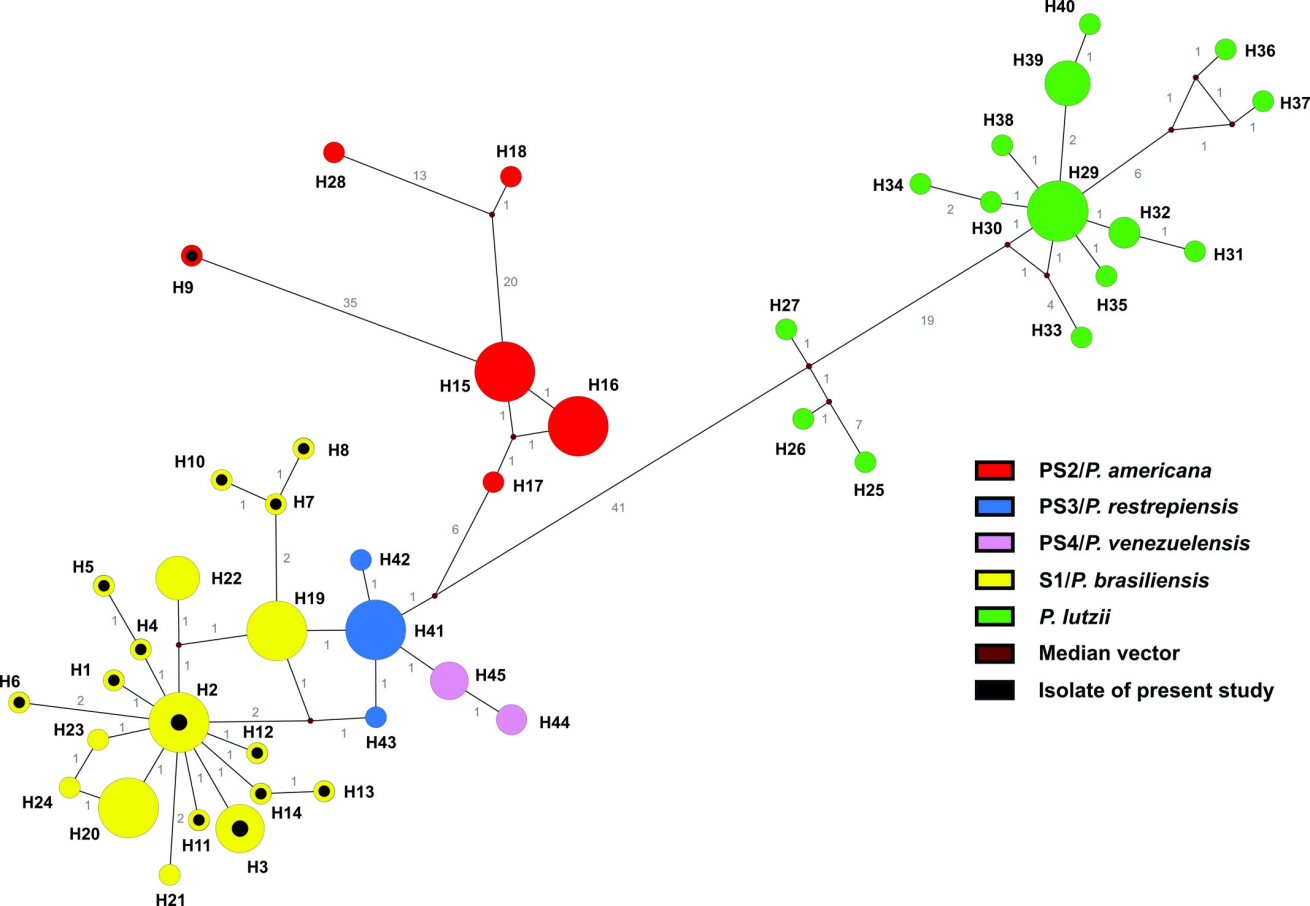
Median-joining haplotype network of 174 *Paracoccidioides* spp. based on concatenated nucleotide sequences of the loci GP43 + ARF. The especies are diferenciated by colors as follow: red PS2/*P*. *americana*, blue PS3/*P*. *restrepiensis*, pink PS4/*P*. *venenzuelensis*, yellow S1/*P*. *brasiliensis* and green *P*. *lutzii*. Each circle represents a unique haplotipe (H), and the circumference is proportional to haplotype frequency (H2: 55 isolates; H41:77: H29:14; H19: 7; H15: 8; H16: 7; H20: 8; H3: 5; H39: 4; H22: 4; H45: 3; H44: 2; H32: 2 and the remaining is composed by one isolate each). Brown dots (median vectors) are hypothetical missing intermediates.

**Fig 3:**
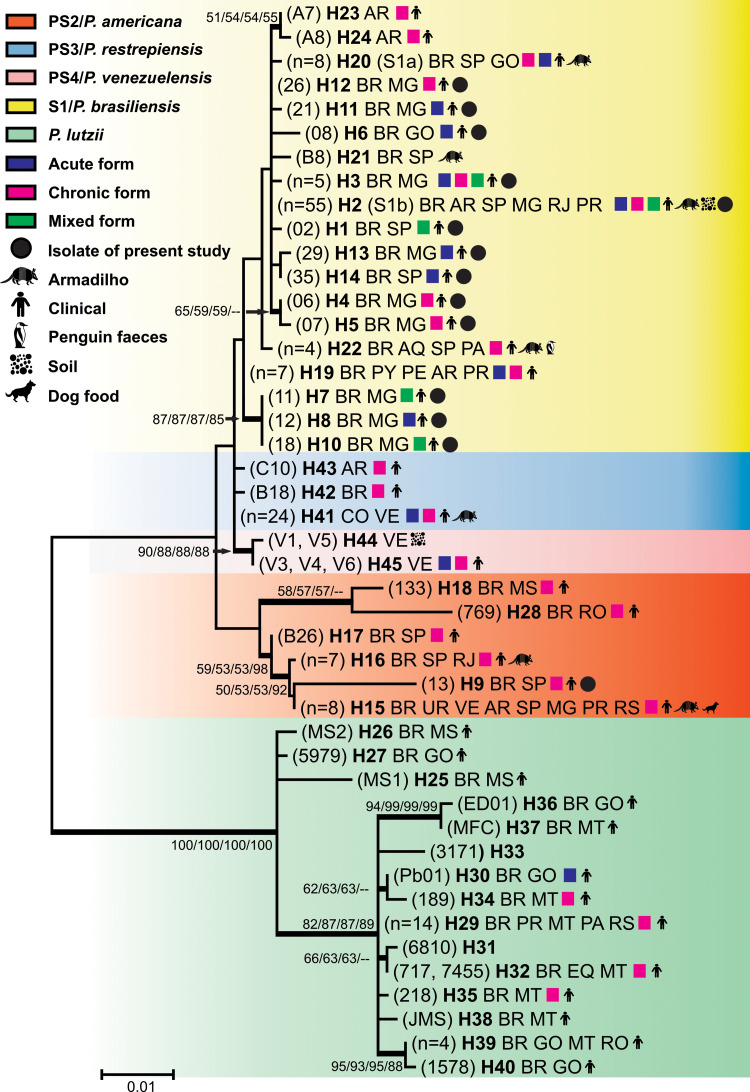
Phylogenetic analysis of 45 haplotipes of *Paracoccidioides* spp. The phylogenetic analysis was inferred by the maximum likelihood (ML), neighbour-joining (NJ), Maximum Parsimony (MP), and unweighted pair group methods with arithmetic mean (UPGMA) using the concatenated sequences of loci GP43 + ARF. These concatenated sequences presented 45 different haplotypes. The tree with the highest log likelihood (-2206.5961) is shown. The tree is drawn to scale, with branch lengths measured in the number of substitutions per site. The analysis involved 45 nucleotide sequences. The codon positions included were 1st+2nd+3rd+Noncoding. There were a total of 814 positions in the final dataset. Evolutionary analyses were conducted in MEGA7. The numbers at each branch indicate bootstrap values *>*50% based on 1,000 replicates by each of the three (ML/NJ/MP/UPGMA) algorithms which presented similar topologies. The five especies of *Paracoccidioides* are marked with different colors. The haplotypes are described according to the names or numbers of isolates which compose the haplotype. When the number of isolates is less than or equal to three all isolates are described in parentheses. When the number is more than three is cited the number of isolates that composed the haplotype followed by haplotipe number in bold (**H**), country from which isolates are originated, states from origin, type of clinical presentation and/or source of isolate. The countries where the isolates were recovered are abbreviated according to the alfa-2 code of ISO 3166 ± 1. AR: Argentina, Antartica: AQ, BR: Brazil, CO: Colombia, EQ: Equador, PY: Paraguai, PE: Peru, UY: Uruguay, VE: Venezuela.

Among the 174 isolates evaluated (Tables 2 and S1), the GP43 was the most variable locus. Furthermore, the vast majority (33/35) of allelle types (ATs) of this locus were species-specific. Of these, 11 (AT1, AT3-AT12) were present exclusively in isolates from the present study (Fig 1B). The ARF locus also exhibited ATs exclusive to *P*. *lutzii* (AT11-AT14), *P*. *americana* (AT4 and AT10) and AT5 and AT3 were found exclusively in isolates from the present study (S1 Table and Fig 1A). The concatenated loci (GP43 + ARF) showed 45 haplotypes (H) (Figs 1C and 2). Of these, 19 in S1/*P*. *brasiliensis* (13 exclusive to the present study), 15 in *P*. *lutzii*, six in PS2/*P*. *americana* (one exclusive to the present study, H9), three in PS3/*P*. *restrepiensis* and two in PS4/*P*. *venezuelensis* (Figs 2 and 3).

**Table 2:** DNA polymorphisms in different groups to loci GP43 + ARF in 174 *Paracoccidioides* spp. isolates.

(number of isolates)	Length	S	*π*	K	h	Hd	D	FD	FF	FS	Theta-w	Rm	PHI	References
ARF (174)	376	24	0.01155	4.216	12	0.718	-0.0903	-1.98850	-1.47232	2.537	4.182	0	1.0	[12,13,27,35–47,49]
GP43 (174)	438	88	0.04068	16.55	33	0.843	0.0677	-1.07524	-0.66457	3.110	15.333	5	0.8297	[12,13,27,35–47,49]
All isolates (174)	814	115	0.02707	21.032	45	0.866	-0.0334	-1.73709	-1.13430	1.883	20.038	**6**	**<0.0001**	[12,13,27,35–47,49]
S1/*P*. *brasiliensis* (78)	791	79	0.00592	4.613	20	0.655	**-2.3272**	-1.73279	**-2.37732**	-2.600	15.349	**3**	**<0.0001**	[12,36,37,39–41,43,44]
PS4/*P*. *venezuelensis* (5)	779	1	0.00077	0.600	2	0.600	1.2247	1.22474	1.15728	0.626	0.480	0	&	[40,42,46]
PS3/*P*. *restrepiensis* (26)	779	2	0.00020	0.154	3	0.151	-1.5131	-2.20378	-2.31853	-2.176	0.524	0	&	[12,35,37,39,40,45,47,49]
PS2*/P*. *americana* (18)	811	26	0.00436	3.399	5	0.680	**-2.2623**	**-3.26020**	**-3.44508**	2.101	7.559	0	&	[12,13,36,38–40]
*P*. *lutzii* (29)	788	24	0.00370	2.906	12	0.649	**-1.8720**	-1.76207	-2.11326	-3.506	6.111	0	1.0	[13,27,39,50]
Present Study (23)	805	32	0.00553	4.308	11	0.866	**-1.9830**	**-3.02217**	**3.16250**	-1.714	8.670	0	1.0	This study
Clinical (67)	805	42	0.00614	4.779	24	0.864	-1.5873	**-2.73456**	**-2.74854**	-7.397	8.797	3	0.9076	[12,13,35–37,39,40,42,45,47–49]
Armadillo (13)	779	18	0.00467	3.641	6	0.769	1.5797	-1.88677	-2.06207	0.415	5.800	0	1.0	[35–37]
Other origin (4)	779	18	0.01220	9.500	4	1.000	-0.3313	-0.33131	-0.34183	0.274	9.818	0	1.0	[12,38,39,43,44,46]
Argentina (8)	779	6	0.00312	2.429	5	0.857	0.2306	0.34334	0.35004	-0.731	2.314	0	1.0	[12,39,41]
Brazil (49)	805	40	0.00677	5.271	19	0.873	-1.5080	-2.28123	-2.38595	-3.842	8.971	2	0.8407	[12,13,27,36–40,43,47,48]
Colombia (20)	779	0	0	0	1	0	-	-	-	-	-	0	&	[12,35,39,40,45,47,49]
Venezuela (21)	779	13	0.00582	4.533	3	0.733	-1.2457	-1.25472	-1.35825	2.726	5.693	0	&	[36,39,40,42,46]
Acute (14)	780	15	0.00454	3.538	10	0.848	-1.0186	-1.45921	-1.53548	-3.859	4.717	1	0.3694	[13,39–41]
Chronic (64)	803	37	0.00659	5.131	19	0.945	-1.2424	-2.02798	-2.07107	-2.777	7.825	2	0.9008	[12,13,35,36,39–42,45,47,49]
Mixed (5)	780	7	0.00488	3.800	4	0.900	0.9127	0.91278	0.95142	0.051	3.360	0	1.0	This study
MG (20)	779	20	0.00459	3.578	8	0.821	-1.5088	-2.05288	-2.02985	-2.145	3.579	1	0.5885	[12,38,39,43]
SP (22)	779	22	0.00904	7.039	10	0.840	-0.7638	-1.06207	-1.13483	0.639	8.504	2	0.6419	[12,36,40]
GO (7)	779	14	0.04401	34.286	7	0.097	0.61280	0.98833	1.00144	0.247	30.612	0	1.0	[13,40]
MT (14)	786	15	0.00273	2.143	7	0.692	**-2.2252**	**-2.94203**	**-3.14875**	-1.862	4.717	0	&	[13]
PR (5)	779	10	0.04650	37.000	4	0.900	1.40740	1.40740	1.52696	3.867	31.200	0	1.0	[39,40]
Other Brazilian States # (12)	779	10	0.04656	36.273	10	0.980	1.63627	0.92347	1.26761	1.517	25.829	1	**<0.0001**	[12,13,27,37,39,50]

S–number of polymorphic sites; *π*–nucleotide diversity; k–average number of nucleotide differences; h–number of haplotypes; Hd–haplotype diversity; D–Tajima’s D; FD–Fu and Li’s D; FF–Fu and Li’s F; Fs–Fu’s Fs; *–p<0.05; Rm–Minimum number of recombination events; Theta w–Theta (per sequence) from S; The DNA polymorphism was evaluated excluding sites with gaps. The repeated sequence types from different regions are not included in the total number; Phy–Pairwise Homoplasy Index; &–there are too few informative characters to use the Phi Test as implemented here; Other origins = dog food + soil + penguin faeces; #–four or more sequences are needed to compute the tests. Thus, in this analysis, isolates from the Brazilian States of Mato Grosso do Sul (MS), Rondônia (RO), Pará (PA), Rio Grande do Sul (RS) and Rio de Janeiro (RJ) were used. Minas Gerais State (MG), São Paulo State (SP), Góias State (GO), Mato Grosso State (MT) and Pará State (PR).

Table 2 shows a summary of the comparison between different groups of *Paracoccidioides* spp. from the present study and from other different studies from elsewhere [12,13,27,35–47]. In this comparison the most polymorphic species was S1/*P*. *brasiliensis* [12,36,37,39–41,43,44] with 19 haplotypes, haplotype diversity (Hd) of 0.655 and nucleotide diversity (K) of 4.61 whereas the least polymorphic was PS4/*P*. *venezuelensis* (two haplotypes, HD 0.6 and K 0.6) [40,42,46]. The isolates from other sources (dog food, soil and from penguin faeces) [12,37,38,42,43,45] were more variable than the clinical and armadillo [35–37] ones. In general, isolates from Brazil [12,13,27,36–40,43,47,48] were more polymorphic than those from other countries [12,35,36,39–42,45–47,49] (Hd = 0.857–0.0 and π = 0.00582–0.0). Within Brazil, the most polymorphic isolates were those from SP [12,36,40] (Hd = 0.933 and π = 0.00872). The isolates from patients with the chronic clinical form [12,13,35,36,39–42,45,47,49] (Hd = 0.945 and π = 0.00659) were also more polymorphic than those obtained from patients with the other clinical forms [13,39–41] (Hd = 0.848–0.900 and π = 0.00454–0.00488) (Table 2).

The 23 isolates herein evaluated proved to be quite polymorphic when compared with isolates from other Brazilian regions [12,13,27,36–40,43,47,48] and other countries [12,35,36,39–42,45–47,49]. They were mostly S1/*P*. *brasiliensis* 22 (95.66%), with only one (4.34%) PS2/*P*. *americana* (isolate 13, H9). These isolates had 14 different haplotypes, the majority (12/14) consisting of only one isolate and 10 were exclusive to the present study, nine in S1/*P*. *brasiliensis* (H1, H4, H5, H6, H7, H8, H10, H12 and H14) and one in PS2/*P*. *americana* (H9). Six isolates of S1/*P*. *brasiliensis* showed 100% identity with the reference strain B17 (S1b, H2) and only one nucleotide difference from the other six haplotypes (H1, H3, H4, H11, H12 and H13) (Figs 2 and 3). Among these isolates, a group consisting of three isolates (H7, H8 and H10) presented a monophyletic grouping, clinical origin and all isolates from Minas Gerais State.

The neutrality tests Tajima’s D, Fu & Li’s D, Fu & Li’s F, and Fu’s Fs showed evidence of purifying selection or population expansion for the 23 isolates from the present study and for those from PS2/*P*. *americana*. Different recombination tests (Watterson estimator (theta), Rm and PHI) suggest the hypothesis of recombination in populations composed of all isolates, S1/*P*. *brasiliensis* and isolates from other states (Table 2).

The haplotype with the highest number of isolates was H2, which contains 54 S1/*P*. *brasiliensis* isolates from different states of Brazil and Argentina. These isolates had different origins (clinical, armadillo and soil). The reference isolate B17 (Pb18) of S1b/*P*. *brasiliensis* was also included in this haplotype. The haplotype with the second highest number of isolates was H41 with 24 S1/*P*. *brasiliensis* isolates of clinical and armadillos origin from Venezuela and Colombia (Fig 3).

Most haplotypes have isolates from clinical origin 41/45 (91%). Haplotypes with isolates exclusively from armadillo (H21/isolate B8) and soil (H2 and H44) were also found, within the latter haplotype (isolates V1 and V2) only described in Venezuela. Isolates from penguin feces and dog food were described only in one haplotype each, H22 and H15, respectively. Most 36/45 haplotypes (80%) are from Brazil. However, haplotypes exclusive to Argentina (H23, H24 and H43) and Venezuela (H44 and H45) were also found. The haplotype distribution by states of Brazil shows that Minas Gerais presented the highest number of haplotypes (11), of which nine were exclusive to this state (Fig 3).

## Discussion

The epidemiological characterization of species of the *Paracoccidioides* genus includes the genetic analysis of the isolates which pointed out the potential relation with their geographic distribution, clinical presentation, therapeutic response and preference for hosts, among others, as it has been described for others fungi that cause disease in humans [51].

In the epidemiological context and natural history of PCM, important factors such as frequent migration of individuals for work reasons and the long latency period of the infection make it difficult to define the exact location where they are infected and the correct association of the identified genotype with the geographic location where the patient gets sick [14,52].

Herein 23 isolates of *Paracoccidioides* spp. obtained from patients with various clinical forms of PCM were evaluated. The molecular characterization of these isolates through the sequencing of the loci GP43 and ARF, showed predominance of S1/*P*. *brasiliensis* (22/23). This finding is in agreement with other authors who reported the high occurrence of this phylogenetic species in South America and Brazil, mainly in the Southeast and South states, where PCM is highly endemic [4,52–54].

In accordance with the taxonomic evolution, S1/*P*. *brasiliensis* was proposed to be constituted by two lineages, S1a and S1b [11], which was later endorsed by other authors [18,52,55]. The S1b lineage was associated with most isolates of *Paracoccidioides* spp. and it is considered by different authors as the most recombinant and variable lineage [11,56,57].

The analysis of the GP43 and ARF sequences allowed to observe that the majority of the isolates in the present study presented complete identity or a few nucleotides difference from isolates of S1b type. The predominance of S1b lineage helps explain the high genetic variability found that even with a small number of isolates it was higher than that observed for the isolates from other Brazilian states and Latin American countries where the PCM is endemic [11,52,55].

The isolate identified as PS2/*P*. *americana* (isolate 13, H9) was recovered from a lung fragment culture from a 42-year-old male patient, born in Ribeirão Preto, São Paulo State, recently diagnosed with HIV infection and who simultaneously presented PCM in its chronic clinical form. Isolates of this species have been previously described in different states of Brazil, Venezuela, Uruguay and Argentina and most of them were obtained from patients with the chronic form of PCM [14,39,52,58].

PS2/*P*. *americana* was recovered from armadillos (*Dasypus novemcinctus*) in different places [18,35] and from a female Doberman dog with generalized lymphadenomegaly [14,58,59]. It has been suggested that this species could be less virulent than S1/*P*. *brasiliensis*, because it was recovered from patients with PCM mild clinical forms [60]. However and in line with other authors, some evidence suggestthat PS2/*P*. *americana* can present virulence similarly to that described for S1/*P*. *brasiliensis* [58,61].

The genomic sequencing of PS2/*P*. *americana* exhibited a lower frequency of introgressions and genetic exchanges when compared to other species of the *P*. *brasiliensis* complex [55]. Isolates 769 and 133 clustered polyphyletically with PS2/*P*. *americana* isolates (Fig 3). About the isolate 769, genomic peculiarities and allele sharing between S1/*P*. *brasiliensis* and PS2/*P*. *americana* were described suggesting that it can correspond to a hybrid or to the presence of ancestral polymorphisms in its genome [13,14]. This fact could be favored by the overlapping of ecological niches between the two species and by the current evidence of sexual reproduction in the genus *Paracoccidioides* [62,63].

When evaluated by sequences of ARF+GP43 [58], the isolate 769 was also grouped with S1/*P*. *brasiliens*is isolates and by use of different loci with *P*. *lutzii* isolates [13]. Moreover, the analysis with another set of isolates, this isolate was grouped with *P*. *lutzii* using the ARF sequences and with *P*. *brasiliensis* using the GP43 sequences [27]. Taken together, these findings may partly explain why this isolate clustered polyphyletically into PS2/*P*. *americana* in the present study.

The GP43 locus presented the highest polymorphism and the best ability to discriminate among the species, with most ATs unique to a single species. This locus encodes the glycoprotein GP43, considered the immunodominant epitope used for the diagnosis of PCM and the pivotal molecule for the identification of *Paraccoccidiodes* spp. [64,65].

The PbGP43 gene is composed of two exons separated by a 78 bp intron and apparently has a single copy [65]. The variability in the sequence of exon 2 has been described in different isolates since the 1990s, when the first correlations among these sequences with the origin of the isolates and virulence in animal models were described for the first time [65,66]. Studies of this locus with more isolates and with the insertion of more locus allowed to subsidize the definition of the concept of phylogenetic species of *Paracoccidioides* [12,57].

The ATs 20 and 22 of GP43 were shared by S1/*P*. *brasiliensis* and *P*. *lutzii* in the present study. The AT20 was found in MS1 and ED01 isolates from clinical origin from Mato Grosso do Sul and Goiás, respectively. However the MS1 has already been defined as *Paracoccidioides* sp. [50], *P*. *lutzii* [58,67] and S1/*P*. *brasiliensis* [11,55], while ED01 was characterized as *P*. *lutzii* [11,58]. Similarly, AT22 was found in EPM104 and 5979 isolates from clinical origin from Paraná and Goiás, respectively. The latter isolate was also classified in different ways as *Paracoccidioides* sp. [50] e *P*. *lutzii* [16,58], while EPM104 was characterized as *P*. *lutzii* [39,58].

Isolates MS1, MS2 and 5979 grouped with *P*. *lutzii* isolates in a polyphyletic manner (Fig 3) similar to that observed by Macedo et al. 2019 [58]. The genetic diversity of these isolates could be explained by the lack of consensus on their identification and for sharing ATs between *P*. *lutzii* and S1/*P*. *brasiliensis* as evidenced by other authors and herein corroborated [16,27,39,58].

The sharing of ATs could also help to explain the non-differentiation of the species and/or their polyphyletic origin in some of the analyzes carried out. The possibility of disagreement in the separation by phylogenetic species is described in the genealogic concordance for phylogenetic species recognition (GCPSR). This technique was used to differentiate species from several fungal genera and to define that different genetic loci may present different genealogies within the same species due to a recombination process. However, the genealogy of the different loci must be concordant within the same species due to effects of genetic isolation and drift [13,15,68]. Despite a small number of isolates herein evaluated, it was possible to identify a group of three isolates with different haplotypes (H7, H8 and H10), monophyletic grouping, clinical origin and from patiens from Minas Gerais State. Previously, morphological differences between *Paracoccidioides* species and their corresponding geographic area had been already described [27,50,56,57]. Additionally, differences in virulence between species [69,70] and the presence of distinct genetic profiles with variable capacity to infect mice have also been reported [71]. However, the correlation between clinical isolates and the geographic origin of patients must be interpreted with caution, as these isolates may have been acquired in regions different from those where the patient originated or was diagnosed with PCM [72].

Although the geographic region of the patients with PCM herein evaluated is borderline to the areas where *P*. *lutzii* has been described, none of the isolates was characterized as such. Similarly, in the Southeast region of Brazil, only one isolated of this species was reported among 46 clinical and four environmental isolates evaluated [52]. Another study with 40 clinical and environmental samples from different geographic origin in South America found *P*. *lutzii* in 20% of the samples, recovered from patients from the Midwest region of Brazil [39], where case-series reports of this species are sparse and still incipient regarding the geographic mapping of its distribution. The natural habitat of this species has not been well elucidated and has not been isolated from armadillos yet [53,73–75].

Despite the small number of isolates included, the data herein presented confirm the predominance of S1b/*P*. *brasiliensis* in Minas Gerais as already described for other states from the Southeast Region of Brazil. Moreover, a significant intraspecific variability and a potential correlation of the molecular profile with the clinical form and geographic origin of patients with PCM can be observed.

Thus, the evaluation of a larger number of isolates together with the analysis of sequence data deposited in GenBank from other Brazilian regions and Latin American countries where PCM is endemic can contribute to expand the plotting of the geographic distribution of *Paracoccidiodes* spp. and to elucidate the hypotheses about the correlation of their molecular profile with the PCM clinical forms, virulence, therapeutic response, host preference, among others.

## Supporting information

S1 Table General data on *Paracoccidioides* spp. evaluated in this study. (XLSX)
